# Epidural Electrical Stimulation of the Lumbosacral Spinal Cord Improves Trunk Stability During Seated Reaching in Two Humans With Severe Thoracic Spinal Cord Injury

**DOI:** 10.3389/fnsys.2020.569337

**Published:** 2020-11-19

**Authors:** Megan Gill, Margaux Linde, Kalli Fautsch, Rena Hale, Cesar Lopez, Daniel Veith, Jonathan Calvert, Lisa Beck, Kristin Garlanger, Reggie Edgerton, Dimitry Sayenko, Igor Lavrov, Andrew Thoreson, Peter Grahn, Kristin Zhao

**Affiliations:** ^1^Assistive and Restorative Technology Laboratory, Department of Physical Medicine and Rehabilitation, Rehabilitation Medicine Research Center, Mayo Clinic, Rochester, MN, United States; ^2^Mayo Clinic Graduate School of Biomedical Sciences, Mayo Clinic, Rochester, MN, United States; ^3^Department of Neurobiology, University of California, Los Angeles, Los Angeles, CA, United States; ^4^The Centre for Neuroscience and Regenerative Medicine, Faculty of Science, University of Technology Sydney, Ultimo, NSW, Australia; ^5^Department of Neurosurgery, Center for Neuroregeneration, Houston Methodist Hospital, Houston, TX, United States; ^6^Department of Neurology, Mayo Clinic, Rochester, MN, United States; ^7^Institute of Fundamental Medicine and Biology, Kazan Federal University, Kazan, Russia; ^8^Department of Neurologic Surgery, Mayo Clinic, Rochester, MN, United States; ^9^Department of Physiology and Biomedical Engineering, Mayo Clinic, Rochester, MN, United States

**Keywords:** spinal cord injury, epidural spinal electrical stimulation, modified functional reach test, reach distance, trunk stability, neuromodulation, neurorehabilitation, paralysis

## Abstract

**Background:** Quality of life measurements indicate that independent performance of activities of daily living, such as reaching to manipulate objects, is a high priority of individuals living with motor impairments due to spinal cord injury (SCI). In a small number of research participants with SCI, electrical stimulation applied to the dorsal epidural surface of the spinal cord, termed epidural spinal electrical stimulation (ES), has been shown to improve motor functions, such as standing and stepping. However, the impact of ES on seated reaching performance, as well as the approach to identifying stimulation parameters that improve reaching ability, have yet to be described.

**Objective:** Herein, we characterize the effects of ES on seated reaching performance in two participants with chronic, complete loss of motor and sensory functions below thoracic-level SCI. Additionally, we report the effects of delivering stimulation to discrete cathode/anode locations on a 16-contact electrode array spanning the lumbosacral spinal segments on reach distance while participants were seated on a mat and/or in their wheelchair.

**Methods:** Two males with mid-thoracic SCI due to trauma, each of which occurred more than 3 years prior to study participation, were enrolled in a clinical trial at Mayo Clinic, Rochester, MN, USA. Reaching performance was assessed, with and without ES, at several time points throughout the study using the modified functional reach test (mFRT). Altogether, participant 1 performed 1,164 reach tests over 26-time points. Participant 2 performed 480 reach tests over 17-time points.

**Results:** Median reach distances during ES were higher for both participants compared to without ES. Forward reach distances were greater than lateral reach distances in all environments, mat or wheelchair, for both participants. Stimulation delivered in the caudal region of the array resulted in improved forward reach distance compared to stimulation in the rostral region. For both participants, when stimulation was turned off, no significant changes in reach distance were observed throughout the study.

**Conclusion:** ES enhanced seated reaching-performance of individuals with chronic SCI. Additionally, electrode configurations delivering stimulation in caudal regions of the lumbosacral spinal segments may improve reaching ability compared to rostral regions.

## Introduction

Traumatic spinal cord injury (SCI) can drastically disrupt mobility and change the way individuals interact with their surroundings, prompting adaptations to maximize the independent performance of activities of daily living (ADLs). While in a seated position, impairment of trunk and leg muscle activation after SCI leads to an inability to maintain the position of the spine, pelvis, and hips when challenged against gravity. Thus, individuals with SCI have a significantly diminished ability to reach forward, or laterally, from a seated position, as well as a reduced capability to perform movements that are dependent upon motor control of the trunk and postural muscles (Chen et al., 2003).

Sensorimotor functional impairment in individuals with SCI inevitably leads to increased risk of fall-related injuries when performing ADLs, and results in a poor posture that compromises shoulder stability (Cloud et al., 2017) and skin integrity (King et al., 2008). Undoubtedly, individuals with tetraplegia struggle with postural instability more than individuals with paraplegia due to a greater dysfunction of trunk musculature (Chen et al., 2003; Milosevic et al., 2015). Regaining trunk stability, which is one of the top priorities identified by those living with SCI, would reduce the risk of fall-related injury and increase the independent performance of ADLs (Brown-Triolo et al., 2002; Anderson, 2004). Therapeutic approaches to address trunk stability typically focus on neuromuscular re-education of the trunk and hip muscles through task-specific balance training (Boswell-Ruys et al., 2010; Tse et al., 2018). Trunk stability can also be gained through compensatory mechanisms such as complex seating systems that are tailored to fit the individual and attach to their wheelchair (Curtis et al., 1995).

Neuromuscular electrical stimulation (NMES) is an intervention that induces motor activation patterns that mimic neurologically intact functional performance with an overarching goal of leveraging intrinsic neuroplasticity to retrain impaired neurocircuitry and improve function in individuals with upper motor neuron damage. Over the past several decades, NMES has been identified as a reliable intervention to improve trunk stability and is suggested as a standard of care along with therapeutic exercise after SCI (Ho et al., 2014; Bergmann et al., 2019). The application of NMES during functional tasks *via* skin surface or implanted stimulating electrodes, described as functional electrical stimulation (FES), has been shown to improve trunk stability and seated posture during reaching tasks for individuals with SCI (Kukke and Triolo, 2004; Triolo et al., 2013a; Bergmann et al., 2019). However, the magnitude of electrically stimulated muscle activation is modest compared to that of the non-injured population under typical physiological conditions (Collins, 2007; Triolo et al., 2013a). Additionally, the efficacy of FES is limited by neurophysiological properties of directly activating peripheral components of neuromuscular circuitry, which is thought to preferentially activate fatigable motor units at lower stimulus intensities than fatigue-resistant motor units (Henneman et al., 1965; Boom et al., 1993; Riess and Abbas, 2001; Godfrey et al., 2002; Popovic et al., 2002). Consideration of spinal cord stimulation could minimize the issue of muscle fatigue of direct NMES allowing longer durations of stimulation enabled functions.

Over the last decade, transcutaneous spinal electrical stimulation and epidural spinal electrical stimulation (ES) have emerged as promising approaches that facilitate spinal sensorimotor circuits in a manner that produces a more physiological activation pattern compared to FES (Sayenko et al., 2014, 2015; Gerasimenko et al., 2008, 2015a,b; Minassian et al., 2016a; Grahn et al., 2017; Hofstoetter et al., 2018). Additionally, in contrast to the use of FES as a neuroprosthetic technology, evidence suggests spinal stimulation engages spared sub-functional connections that span the injury site to restore volitional control over stimulation-enabled motor activity (Minassian et al., 2016b; Ievins and Moritz, 2017; Calvert et al., 2019a; Cho et al., 2019). For example, postural stability and ability to regain balance during self-initiated perturbations within a single session have been described through the use of transcutaneous spinal electrical stimulation in humans with motor complete (*N* = 6), as well as motor incomplete (*N* = 2), SCI (Rath et al., 2018); however, ES-enabled trunk stability and reaching ability while seated have not been described in detail. The underlying mechanisms through which ES, as well as transcutaneous spinal electrical stimulation, enables functional gains are thought to involve the facilitation of a “central state of excitability” within spinal networks that reside below the level of SCI (Taccola et al., 2018). Following the described theory, ES could potentially result in similar improvements in trunk stability to those described during transcutaneous electrical spinal stimulation. Optimizing stimulation parameters for task-specific activities relies on multiple different variables, including electrode location and voltage intensity. Localized activation of the rostral electrodes primarily activates proximal muscles, whereas localized activation of caudal electrodes activates predominately distal muscles (Sayenko et al., 2014; Calvert et al., 2019a).

We previously demonstrated that ES in combination with task-specific training, which we defined as multimodal rehabilitation (MMR), likely facilitates reorganization of the supraspinal-spinal connectome to recover lost functions following SCI (Gill et al., 2018). Similarly, multiple reports have shown that over several months of MMR sessions, performed multiple days per week, individuals with SCI achieved improvements in standing performance in the presence of ES (Harkema et al., 2011b; Rejc et al., 2015, 2017; Grahn et al., 2017) as well as restoration of independent weight-bearing stepping activity (Angeli et al., 2018; Gill et al., 2018; Wagner et al., 2018) and trunk stability (Angeli et al., 2018). Here, we describe the effects of ES on seated reaching ability in two individuals with chronic motor and sensory complete paraplegia following SCI. Secondly, we describe seated reaching outcomes produced by localizing active electrode configurations within the rostral, and caudal regions of the implanted electrode array.

## Materials and Methods

### Participant Descriptions

At the time of study enrollment, participant 1 was a 26-year-old male who sustained a traumatic SCI at the T6 vertebral level 3 years prior and was diagnosed as American Spinal Injury Association Impairment Scale-A (AIS-A; i.e., complete loss of motor, sensory, and autonomic functions below the level of injury). We previously reported lower extremity motor functions that were restored using ES, such as standing and stepping, in participant 1 (Grahn et al., 2017; Gill et al., 2018; Calvert et al., 2019a).

At the time of enrollment, participant 2 was a 37-year-old male who sustained a traumatic SCI at the T3 vertebral level 6 years prior and was diagnosed as AIS-A. Together with data generated by participant 1, we previously reported that the participant achieved a step-like movement of his lower extremity using ES while positioned side-lying with his leg suspended in a gravity-neutral position (Calvert et al., 2019a).

Both participants provided written informed consent to conduct experiments described within a study protocol that was approved by the FDA for an investigational device exemption as well as approved by Mayo Clinic’s IRB. For mobility in their personal lives, both participants used rigid frame, self-propelled wheelchairs that were custom-fit to maximize comfort, appropriate posture, and trunk stability.

### ES System and Rehabilitation Paradigm

**Figure 1:**
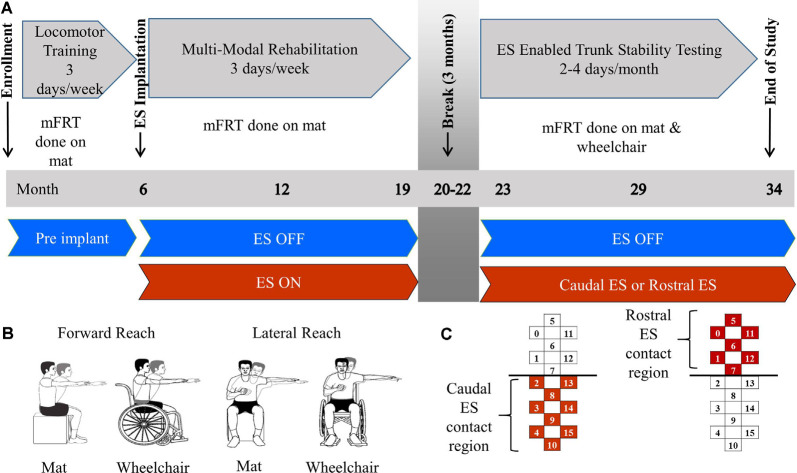
Methods description. Panel **(A)** describes clinical trial timeline including enrollment, time of electrical stimulation (ES) implantation, 3 month break, and end of study. Training focus is described for each phase of the study as well as the environment of each modified functional reach test (mFRT). Panel **(B)** is a pictorial of forward and lateral reaching tasks performed on the mat or wheelchair. Panel **(C)** demonstrates the active electrodes used on the stimulating array for Caudal ES and Rostral ES.

Both participants underwent 6 months of locomotor training (Harkema et al., 2011a; Figure 1A) followed by surgical implantation of a 16-contact epidural spinal electrical stimulation electrode array (Specify 5-6-5, Medtronic, Fridley, MN, USA) at the T11-L1 vertebral region. To refine electrode array alignment to the lumbosacral spinal cord enlargement (i.e., spinal segments L2-S1), intraoperative electromyography was used to record ES-evoked motor potentials from several muscles of the lower extremities, bilaterally (Calvert et al., 2019a). After 3 weeks of rest, each participant performed approximately three sessions of MMR per week for the next 12 months. MMR sessions were comprised of ES parameter adjustment to enable maximum independence during stand, step, and reach training. During the 12 months of MMR, participants were allowed to use a subset of ES parameters outside the laboratory, only if deemed safe by study staff, to perform tasks, such as supine or seated volitional leg movements and standing with appropriate assistive devices (Gill et al., 2018). After 12 months of MMR, each participant took a 3-month break from study-related activities. Then, they performed 12 additional months of MMR sessions and testing, during which they attended two days of laboratory-based activities twice a month, which focused on examining ES-enabled trunk stability and reaching functions.

### Modified Functional Reach Test to Assess ES-Enabled Performance

The modified functional reach test (mFRT) is a clinical assessment used to evaluate reaching performance and provide immediate feedback to participants and study staff (Lynch, 1995). The mFRT was performed 1–2 times per month throughout the study while participants were seated either on a padded, height-adjustable mat or while positioned in their wheelchair. At each recording session, the mFRT was performed with, and without ES, while the participants’ feet were positioned flat on the floor or the footrest of their wheelchair. For safety purposes, a trainer was located in front of the participant to prevent falls if a loss of balance occurred. At the start of each recording, they were instructed to raise one arm to 90 degrees of either shoulder flexion (forward reach) or abduction (lateral reach) with their elbow joint fully extended while maintaining a neutral wrist position and extended fingers (Figure 1B). A meter stick was held horizontally by study staff in proximity to the participant’s finger. Zero distance marked the starting point and maximum reach distance was captured when the participant reached forward or laterally as far as possible while retaining the ability to independently return to their initial, upright seated position. The participant’s uninvolved arm could be used for counterbalance, but not for support while reaching. If the uninvolved arm was used for support, or if trainer assistance was required to return to the initial position, the attempt was not recorded for data analysis, and a subsequent attempt was performed. Three independent reaches were collected for each condition: ES ON and NO ES (e.g., left arm forward, right arm forward, left arm lateral, right arm lateral). The sequence of these four conditions was not standardized across sessions or participants. ES pulse amplitude, width, and frequency, as well as anode/cathode configurations, were adjusted during each testing session with a focus on improving trunk stability. Rostral ES and Caudal ES were defined as localized programs increasing stimulation intensity to facilitate the greatest reaching distance possible. Regional descriptions of the electrodes used (anodes and cathodes) for Rostral ES and Caudal ES are visually provided in Figure 1C. The parameters used for this study were a subset of the ranges that are defined by the ES device manufacturer, which were approved for use in this study by the Mayo Clinic IRB after obtaining an IDE from the FDA. For comparison purposes, Caudal ES and Rostral ES parameters were tracked over months 23–34. During Caudal ES, stimulation intensity and frequency ranges for participant 1 were 2.0–6.5 V and 20–25 Hz with a 210 μs pulse width and during Rostral ES, the same parameter ranges were 4.4–7.8 V and 20 Hz with a 420 μs pulse width. During Caudal ES, the stimulation intensity range for participant 2 was 2.9–3.0 V with a frequency of 20 Hz and pulse width range of 200–400 μs, while during Rostral ES, the stimulation intensity range was 3.8–5.0 V with a frequency of 25 Hz and pulse width of 450 μs.

### Reach Distances Across Clinical Trial Time Points

#### Participant 1

**Figure 2:**
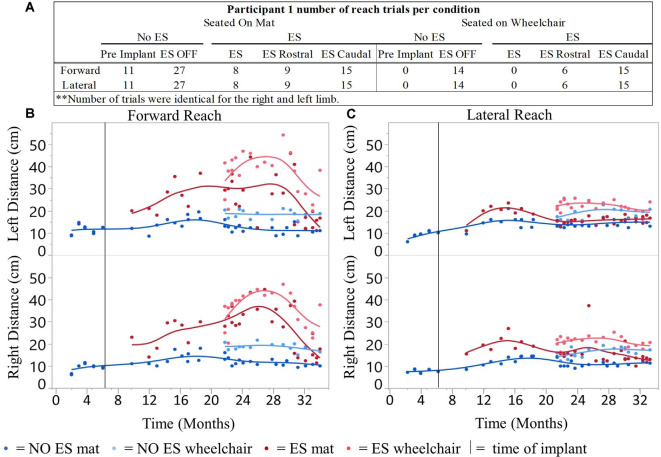
Participant 1 reach distances for all conditions recorded over time. Number of mFRT trials recorded throughout the study demonstrating forward and lateral (right and left equally), through all ES conditions: NO ES and ES. Numbers display trials performed on mat and on wheelchair **(A)**. The average of three trials per day for forward **(B)** and lateral reach **(C)** on mat and wheelchair. Solid vertical line indicates epidural stimulator implantation time point.

For all conditions tested, a total of 1,164 successful reaches were recorded using the mFRT across 26-time points resulting in 388 averaged data points (Figure 2). Out of the 388 averaged data points, 208 represent reaching performance without ES and 180 represent reaching performance with ES.

#### Participant 2

A total of 480 mFRT recordings were collected across 17-time points of the clinical trial. From those recordings, 60 represent reaching performance without ES and 100 represent reaching performance with ES (Figure 6). All mFRT were performed while seated on a mat. During month 22, the participant was withdrawn from the clinical trial due to personal commitments, not due to study-related complications or adverse events.

**Figure 3:**
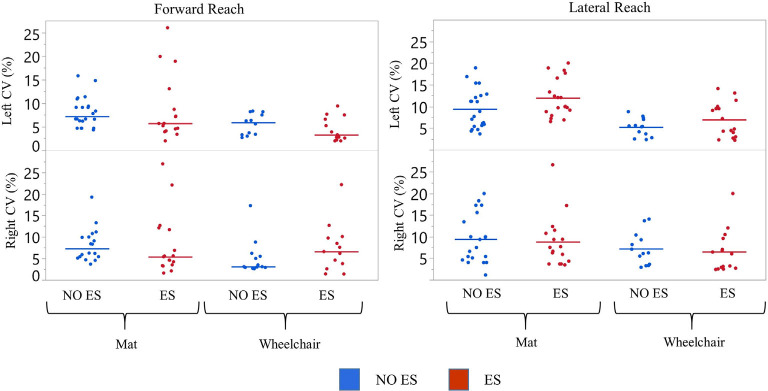
Participant 1 coefficient of variation (CV) of reach scores. The CV was calculated for all reach distances of NO ES (Blue) and ES (Red) for the right and left arm while seated on a mat or a wheelchair. Data represented in a scatter plot with line at the median value.

**Figure 4:**
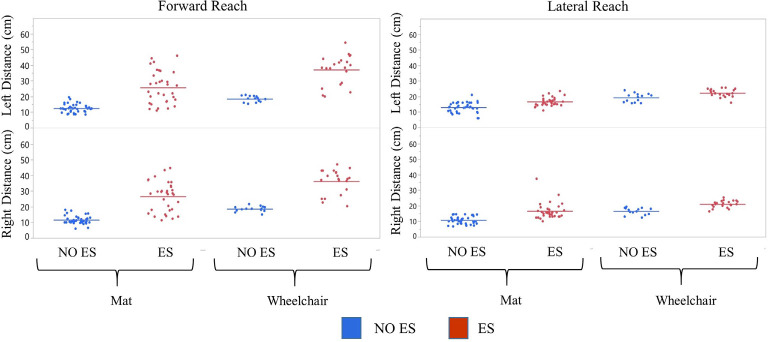
Participant 1 comparison of No ES to ES reach distances. Forward and lateral reach distances during No ES and ES conditions were compared for right and left sides while seated on the mat or the wheelchair. Dots represent the average of three trials for forward and lateral reach and solid horizontal line represents the median of all trials combined.

**Figure 5:**
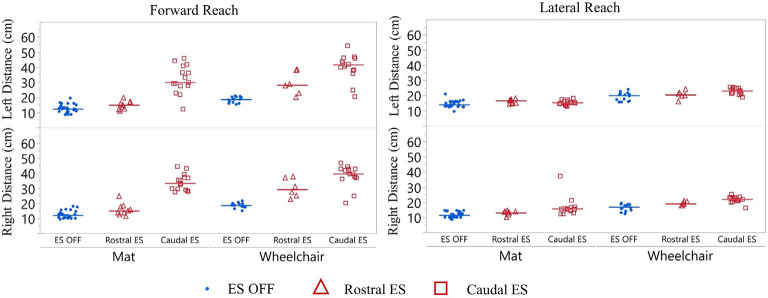
Participant 1 reach distance during three conditions (ES OFF, ES Rostral and ES Caudal) on mat and wheelchair. ES conditions for forward and lateral reach distances reported for right and left sides. Each data point indicates the average of three trials at each test date, blue represents ES off, red triangles represent Rostral ES usage, and red squares represent Caudal ES usage. Each reach direction, forward, lateral, right, and left were performed and reported for mat and wheelchair environments.

**Figure 6:**
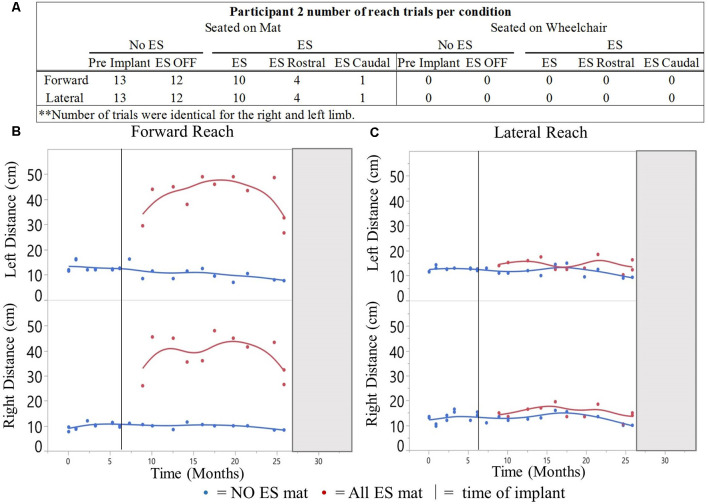
Participant 2 reach distances for all conditions recorded over time. Number of mFRT trials recorded throughout the study demonstrating forward and lateral (right and leftarm), through all ES conditions: NO ES and ES. Numbers display trials performed on mat **(A)**. The average of three trials per day for forward **(B)** and lateral reach **(C)**. Solid vertical line indicates ES implantation time point. Gray box represents when the participant exited the study.

### Data Analysis

Reach distance recordings from three successful, independent reaches were averaged. Averaged reach distances were categorized by reaching limb (right or left), reaching direction (forward or lateral), environment (mat or wheelchair), and ES and NO ES conditions. To evaluate the repeatability of reach distances within each trial, the coefficient of variation (CV) was calculated for each condition tested. The NO ES condition was then subdivided into pre-implant (months 0–6) and ES OFF (months 6–34). The ES condition was then subdivided into Rostral and Caudal ES (months 23–34; Figure 1). Due to non-normal distribution, data were summarized and presented descriptively as median values and interquartile ranges (IQR) calculated using JMP statistical software (SAS, Cary, NC, USA). One group’s value was considered to be notably larger than that of another if medians were different and if more than two-thirds of the data points in the stated lesser-valued group fell below the median of the greater-valued group. Reach distances were calculated and plotted across time according to when mFRT recordings were gathered during the clinical trial. The timing of mFRT recordings is shown as a test date minus enrollment date. For pictorial analysis only, a spline fit was generated for median values across time. Data from participant 1 was analyzed independently from participant 2.

## Results

### Participant 1

#### Variability of Reaching Performance Across Experimental Conditions

Left median forward reach CV values for the mat and wheelchair conditions were higher during the NO ES condition when compared to the ES condition. A small difference in CV was noted for the lateral reaching task (Figure 3). While seated on the mat, median forward reach distance variability was 1.9% higher for the right arm and 1.5% higher for the left arm; while median lateral reach distance variability was 0.9% higher for the right arm and 1.7% lower for the left when comparing the NO ES condition to ES conditions. While seated in the wheelchair, median forward reach distance variability was 3.5% lower for the right arm and 2.6% higher for the left arm while median lateral reach variability was 0.2% higher for the right arm and 0.6% lower for the left arm when comparing the NO ES condition to ES condition. During ES conditions, median forward reach distance variability was 1.2% lower for the right arm and 2.4% higher for the left arm, while median lateral reaching distance variability was 1.5% higher for the right arm and 4.0% higher for the left arm when comparing the mat to wheelchair environment. During the NO ES condition, the median forward reach distance variability was 4.2% higher for the right arm and 1.3% higher for the left arm, while median lateral reaching was 2.2% higher for the right arm and 2.9% higher for the left arm when comparing the mat to the wheelchair environment.

#### Reaching With ES Compared to NO ES

Participant 1 consistently had a higher median forward and lateral reach distance with ES compared to NO ES condition when seated on the mat as well as in the wheelchair. While seated on the mat, ES resulted in greater median forward reaching distances by 17.4 cm (right) and 12.7 cm (left) than NO ES. Additionally, median lateral reaching distances with ES increased by 5.1 cm (right) and 3.3 cm (left), respectively. While seated in the wheelchair, ES resulted in median forward reaching distances that were 19.0 cm (right) and 19.6 cm (left) greater than NO ES, as well as median lateral reaching distances that were 4.2 cm (right) and 2.0 cm (left) greater (Figure 4). In the mat and wheelchair environments, improvements in reach distances, specifically in forward but not lateral reach, resulted in an instantaneous effect when utilizing ES.

##### Reaching While Seated on the Mat Compared to Seated on the Wheelchair

Participant 1 consistently reached farther (forward and laterally) in the wheelchair than on the mat in both ES and NO ES conditions (Figure 4). Reaching with ES while seated in the wheelchair resulted in median forward reaching distances that were 9.3 cm (right) and 13.3 cm (left) greater than reaching while seated on the mat. Similarly, median lateral reaching distances were 5.6 cm (right) and 5.7 cm (left) greater from the wheelchair compared to the mat. Reaching from the wheelchair during NO ES resulted in median forward reach distances that were 7.7 cm (right) and 6.4 cm (left) greater than reaching from the mat. Likewise, median lateral reach distances were 6.5 cm (right) and 7.0 cm (left) greater during NO ES when reaching from the wheelchair as compared to NO ES reaching from the mat.

#### Comparison of Rostral ES, Caudal ES, and ES OFF Conditions

Participant 1 consistently reached farther during forward reach, using Caudal ES, compared to Rostral ES, and ES OFF conditions. Additionally, superior reaching performance during Caudal ES was observed when seated on the mat as well as when seated in the wheelchair (Figure 5).

##### Mat Environment

While seated on the mat, there was a greater difference in reaching distances during forward reaching as compared to lateral reaching. Compared to ES OFF, Rostral ES generated an increase in median forward reach distance of 4.0 cm (right) and 2.7 cm (left), as well as an increase in median lateral reach distance of 2.3 cm (right) and 3.4 cm (left). Compared to ES OFF, Caudal ES resulted in median forward reach distances that were 22.3 cm (right) and 17.7 cm (left) greater and median lateral reach distances that were 5.0 cm (right) and 2.0 cm (left) greater. Compared to Rostral ES, the Caudal ES electrode configuration enabled greater median reach distances of 18.3 cm (right) and 15.0 cm (left) during forward reaching. Rostral ES enabled greater median lateral reaching for the left arm (1.4 cm) whereas Caudal ES enabled greater lateral reaching for the right arm (2.7 cm).

##### Wheelchair Environment

While seated on the wheelchair, the difference in median reach distance was greatest between ES OFF and Caudal ES. Compared to ES OFF, Rostral ES resulted in median forward reach distances that increased by 10.5 cm (right) and 9.5 cm (left), and median lateral reach distances that increased by 2.2 cm (right) and 0.5 cm (left). Compared to ES OFF, Caudal electrode configurations lead to median increases of 21.0 cm (right) and 23.0 cm (left) during forward reaching as well as increases of 5.2 cm (right) and 3.0 cm (left) during lateral reaching. We found that during Caudal ES forward reaching distances were 10.5 cm (right) and 13.5 cm (left) greater than during Rostral ES. Similarly, median lateral reaching distances were 3.0 cm (right) and 2.5 cm (left) greater during Caudal ES compared to Rostral ES.

#### Comparison of Seated Position (Wheelchair vs. Mat)

For all ES conditions, median reach distances from the wheelchair were greater than those performed while seated on the mat (Figure 5). Sitting in the wheelchair, compared to the mat, led to increases in median forward reaching distances of 7.7 cm (right) and 6.4 cm (left) during ES OFF; 14.2 cm (right) and 13.2 cm (left) during Rostral ES, and 6.4 cm (right) and 11.7 cm (left) during Caudal ES. Similarly, median lateral reach distances while seated in the wheelchair were greater than when seated on the mat: 6.1 cm (right) and 6.7 cm (left) during NO ES; 6.0 cm (right) and 3.8 cm (left) during Rostral ES; and 6.3 cm (right) and 7.7 cm (left) during Caudal ES.

### Participant 2

#### Variability of Reaching Performance Across Experimental Conditions

**Figure 7:**
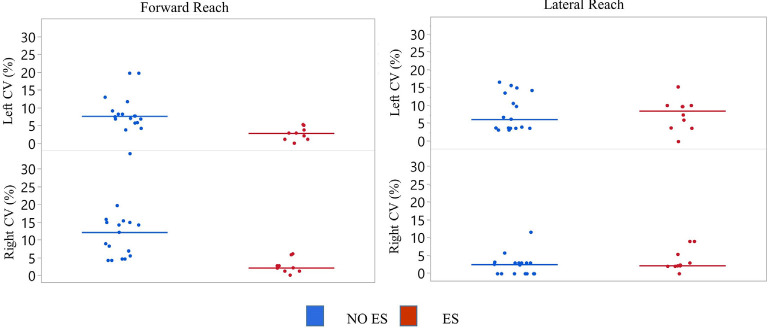
Participant 2 coefficient of variation (CV) of reach scores. The CV was calculated for all reach distances of NO ES (Blue) and ES (Red) for the right and left arm while seated on a mat. Data represented in a scatter plot with line at the median value.

The NO ES condition had slightly higher median CV values when compared to the ES condition during forward reach with the right and left arms and during lateral reach with the right arm (Figure 7). Specifically, the median forward reach distance variability was 4.9% higher for the right arm and 4.5% higher for the left arm when comparing the NO ES condition to the ES conditions. Median lateral reach distance variability during NO ES was 7.6% higher than ES when reaching to the right and 4.8% lower than ES when reaching to the left.

#### Reaching With ES Compared to NO ES While Seated on the Mat

**Figure 8:**
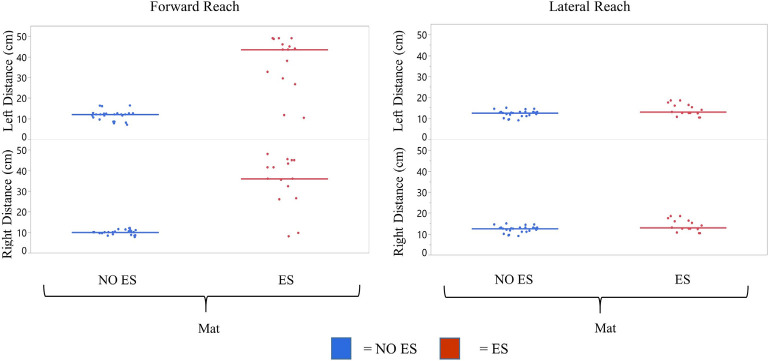
Participant 2 comparison of No ES to ES reach scores. mFRT scores for forward and lateral reach distances in No ES conditions were compared for right and left sides while seated on the mat. Dots represent average of three trials for forward and lateral reach and solid horizontal line represents the median of all trials combined.

Participant 2 demonstrated greater median forward and lateral reach distances with ES compared to NO ES (Figure 8). While seated on the mat, the use of ES resulted in median forward reach distances that were 26.0 cm (right) and 31.5 cm (left) greater than during NO ES. Median lateral reach distances from the mat were 1.5 cm (right) and 0.5 cm (left) greater with ES compared to NO ES. Similar to participant 1, improvement in reach distances, specifically forward, resulted in an instantaneous effect when utilizing ES.

#### Comparison of Rostral ES, Caudal ES, and ES OFF Conditions While Seated on the Mat

**Figure 9:**
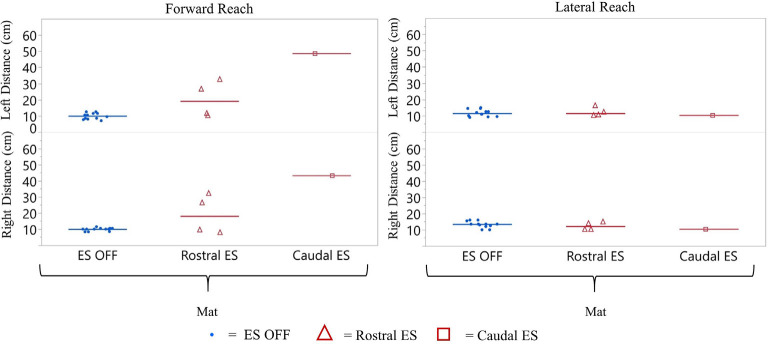
Participant 2 reach distance during three conditions (ES OFF, Rostral ES and Caudal ES contacts) while seated on the mat. ES conditions for forward and lateral reach distances reported for right and left side. Each data point indicates the average of three trials at each test date, blue representing ES off, red triangles represent Rostral ES usage, and red squares represent Caudal ES usage.

Similar to participant 1, participant 2 reached farther forward, with Caudal ES compared to Rostral ES, as well as ES OFF, in the mat environment (Figure 9). Rostral ES resulted in median forward reach distances that were 8.1 cm (right) and 7.7 cm (left) greater than ES OFF. However, during Rostral ES, the median lateral reach distance to the right was 1.3 cm less than ES OFF, and lateral reach distance to the left was 1.0 cm less than ES OFF. Similarly, when compared to ES OFF, Caudal ES median forward reach distances increased by 33.3 cm (right) and 37.2 cm (left) while median lateral reach distances decreased by 3.0 cm (right) and 2.2 cm (left). Likewise, Caudal ES, when compared to Rostral ES, resulted in median forward reach distances that were 25.2 cm (right) and 29.5 cm (left) higher, while median lateral reach distances were 1.7 cm (right) and 1.2 cm (left) lower.

## Discussion

Results from this study demonstrate the feasibility of enhancing trunk stability during seated reaching tasks using lumbosacral ES in humans with chronic SCI. Data from the CV analysis suggests that patients with SCI are repeatable in their forward and lateral reach tests, and reach distances are affected by the ES ON and ES OFF conditions. Results indicate that when ES is enabled, forward reach distances increase, and lateral reach distances remains unchanged. Within the ES condition, caudal stimulation was more effective in improving forward reach distance than rostral stimulation.

### Epidural Spinal Electrical Stimulation Enables Increased Reach Distance

The act of reaching forward or laterally, from a stable seated position to the limit of stability, followed by a return to an upright sitting position can be significantly impaired after a SCI. Impaired reaching ability results in a drastic loss of independence as well as an increase in the risk of injury due to loss of balance and falling. Here, we objectively demonstrated that reach distances instantaneously improved in individuals with SCI in the presence of ES. During ES both participants were able to reach farther in the forward direction using either arm when compared to without ES.

Reach distance variability was dependent upon: (1) the seated environment; (2) the range and direction of mFRT recordings; and (3) the presence of ES compared to NO ES. Results from CV analysis demonstrate that reach distance scores were more repeatable when the participants were sitting in their wheelchair while ES was enabled compared to sitting on the mat with NO ES. Customized manual wheelchair seating systems enhance seated stability by providing individualized support for appropriate pelvic and trunk positioning during ADLs. Although the confidence in reaching ability was subjectively reported by both participants to be greater with ES, the reach distance measurement range was wider, especially during forward reach. When ES was not enabled, reaching ability returned to its original functional state for both participants.

For both participants, ES-enabled improvements in the forward reach distance were notably greater than those observed in lateral reach distance. Forward and lateral reaching requires activation of different muscle groups to achieve direction-specific movement patterns. Factors that typically affect seated posture and could potentially impact lateral reach include pelvic obliquity, presence of scoliosis, and SCI motor asymmetry. We demonstrated that rostral and caudal ES configurations enabled reaching abilities differently. Our findings suggest unique ES configurations may be needed to enable maximum reaching performance in all directions, which may be a critical feature of next-generation ES technologies to successfully translate ES use for ADL performance by individuals with SCI. Additionally, continued investigation of lower extremity activation patterns during reaching and returning to the upright sitting position may provide new insight that can be leveraged during ES configuration optimization to facilitate similar patterns of activation, and in turn, achieve optimal reaching performance with ES. Our results show that the use of ES, specifically caudal ES configurations designed to engage distal leg muscles, generally resulted in greater reach distances when compared to rostral ES configurations outlined in Calvert et al. (2019b).

### Therapeutic Potential of ES

Both participants’ forward reaching ability improved instantaneously with ES compared to without. Of equal importance, these improved reaching abilities with ES were repeatable throughout the study. Our findings suggest that ES provides a therapeutic option for restoring functional trunk stability which is currently untreatable, or at best, marginally improved by long-term, strenuous exercise paradigms (Sliwinski et al., 2020). In addition to enabling supraspinal control over motor functions, ES-enabled motor functions are thought to facilitate a more physiological pattern of motor unit recruitment when compared to currently-available NMES systems (Henneman, 1957; Henneman et al., 1965; Maffiuletti, 2010; Bickel et al., 2011). Furthermore, the magnitude of forward reaching we observed during ES was considerably greater than absolute reach distances reported by others during NMES (Triolo et al., 2013b).

When delivering electrical stimulation to the skin over the spine, which has recently emerged as a promising approach to modulate spinal networks after SCI, direct activation of trunk musculature likely occurs, in a similar manner as FES, in addition to previously described spinal network activation (Hofstoetter et al., 2018; Rath et al., 2018; Sayenko et al., 2019). However, during ES, focal activation occurs within spinal networks, rather than directly activating peripheral components of trunk neuro-musculature. Therefore, the improvements in the seated function we observed may have been achieved engaging multi-segmental spinal networks that span rostrally from the lumbosacral implantation site of the electrode array, which in turn, enabled coordinated activation of muscle synergies across the trunk, hip, and lower limbs to improve the reaching ability.

The evidence presented here demonstrates a critical step toward the restoration of functional motor activity using ES to enhance ADLs in individuals with SCI. Future studies should incorporate full-body biomechanical assessments (e.g., motion capture, electrophysiology, etc.) to better understand dynamic interactions that occur across spinal sensorimotor networks during ES, and the extent to which ES-facilitated spinal networks integrate supraspinal motor control signals across the site of SCI, necessary to generate functional motor outputs, such as improved seated reaching performance. Future studies investigating ES-enabled reaching abilities should include individuals with different classifications of SCI to determine the generalizability of our results. Additionally, we recognize the study reported herein has limitations that challenge generalizing these results, given the heterogeneity of the severity of SCI and limited sample size. In conclusion, our results demonstrate that ES generated instantaneous improvements in seated reaching performance in two individuals with severe, motor, and sensory complete thoracic SCI. Additionally, stimulation delivered in the caudal region of the array resulted in improved forward reach distance compared to stimulation in the rostral region.

## Data Availability Statement

The raw data supporting the conclusions of this article will be made available by the authors, without undue reservation.

## Ethics Statement

The studies involving human participants were reviewed and approved by Mayo Clinic Institutional Review Board (IRB). The participants provided their written informed consent to participate in this study. Written informed consent was obtained from the individual(s) for the publication of any potentially identifiable images or data included in this article.

## Author Contributions

PG, MG, RE, DS, and KZ initiated the project. LB, JC, MG, PG, ML, and DV designed the experiments with contributions from all authors, performed clinical assessments and designed and performed rehabilitation. LB, JC, MG, PG, ML, IL, and DV contributed to stimulation setting refinement. LB, JC, KF, PG, MG, ML, CL, RH, AT, DV, and KZ contributed to data collection, analysis, and interpretation. KF, MG, ML, and RH drafted the manuscript with subsequent contributions from all authors. KZ supervised all aspects of the work. All authors contributed to the article and approved the submitted version.

## Conflict of Interest

RE holds shareholder interest in NeuroRecovery Technologies and holds certain inventorship rights on intellectual property licensed by the Regents of the University of California to NeuroRecovery Technologies. RE holds shareholder interest in spineX Inc. and holds certain inventorship rights on intellectual property licensed by the Regents of the University of California to spineX Inc. RE serves on the scientific advisory board of *in vivo* Therapeutics and ArianRF, and serves as the Chair of the Scientific Advisory board at spineX. The remaining authors declare that the research was conducted in the absence of any commercial or financial relationships that could be construed as a potential conflict of interest.
